# Deep Learning Modeling of Cardiac Arrhythmia Classification on Information Feature Fusion Image with Attention Mechanism

**DOI:** 10.3390/e25091264

**Published:** 2023-08-26

**Authors:** Mingming Zhang, Huiyuan Jin, Bin Zheng, Wenbo Luo

**Affiliations:** 1 Faculty of Science, Beijing University of Technology, Beijing 100124, China; mmzhang@bjut.edu.cn (M.Z.); jinhuiyuan0613@163.com (H.J.);; 2 Zhengzhou Aerotropolis Institute of Artificial Intelligence, Zhengzhou 451162, China

**Keywords:** ECG signal, image conversion, relative position matrix, transfer learning, deep learning

## Abstract

The electrocardiogram (ECG) is a crucial tool for assessing cardiac health in humans. Aiming to enhance the accuracy of ECG signal classification, a novel approach is proposed based on relative position matrix and deep learning network information features for the classification task in this paper. The approach improves the feature extraction capability and classification accuracy via techniques of image conversion and attention mechanism. In terms of the recognition strategy, this paper presents an image conversion using relative position matrix information. This information is utilized to describe the relative spatial relationships between different waveforms, and the image identification is successfully applied to the Gam-Resnet18 deep learning network model with a transfer learning concept for classification. Ultimately, this model achieved a total accuracy of 99.30%, an average positive prediction rate of 98.76%, a sensitivity of 98.90%, and a specificity of 99.84% with the relative position matrix approach. To evaluate the effectiveness of the proposed method, different image conversion techniques are compared on the test set. The experimental results demonstrate that the relative position matrix information can better reflect the differences between various types of arrhythmias, thereby improving the accuracy and stability of classification.

## 1. Introduction

Cardiovascular disease poses a significant threat to human health. As electrocardiography is a key method for representing pathological information, it has been an essential tool for the identification and diagnosis of cardiovascular diseases. For arrhythmia, which is one of the manifestations of cardiovascular disease, various technical difficulties still exist in achieving an accurate diagnosis, and the assistance of modeling electrocardiography is required. The traditional diagnostic technique relies on the clinical experience and feature extraction skills of physicians. However, there are technical challenges in manual diagnosis due to substantial differences in electrocardiograms. In recent years, with the application of deep learning in the medical field, automatic extraction of essential features and the automatic diagnosis of cardiovascular diseases have become research topics.

The extraction of features from ECG signals is a crucial step in the intelligent recognition of cardiovascular diseases. The effectiveness of diagnosis is mainly dependent on the quality of feature extraction. The primary features found in ECG signals commonly include morphological properties, time-frequency features, and statistical information. Qin [1] utilized a discrete wavelet transform to extract morphological features combined with principal component analysis (PCA). An optimized support vector machine (SVM) algorithm was employed to perform a six-classification recognition on the MIT-BIH arrhythmia database. A waveform coding rule was devised to extract multiple morphological features of signals [2], and the combination of a convolutional neural network (CNN) and long short term memory (LSTM) network was used for the multi-classification of ECG signals. Zhu [3] used PCA and dynamic time warping (DTW) to extract multiple features with an employment of improved SVM. Elhaj [4] obtained non-linear properties by utilizing the high-order statistical cumulant of signals. The SVM and neural network were used for the five-class classification of arrhythmias with ten-fold cross-validation. Based on the statistical feature extracted with wavelet packet decomposition, a backpropagation neural network was employed for classification with a genetic algorithm [5]. Zarei [6] extracted non-linear features of signals by utilizing the wavelet transform coefficients with an entropy analysis on the fuzzy entropy, approximate entropy, and conditional entropy.

However, due to the one-dimensional nature of ECG signals, the hidden features are not readily revealed. Thus, traditional deep neural networks have difficulty with automatically extracting effective features, leading to underperformance in classification. In contrast, deep learning techniques have demonstrated excellent performance in image segmentation and classification. As a result, researchers have sought to enhance neural networks by increasing the dimensionality of ECG signals. Consequently, two-dimensional imaging has emerged as a viable direction in automatic arrhythmia classification.

Lu [7] proposed an advanced bidirectional recursive neural network based on residual structure, which was effectively classified for signals using a two-dimensional grayscale spectral image. A discrete cosine residual network algorithm was introduced for recognizing myocardial infarctions, optimizing time-frequency characteristics through a discrete cosine transformation method [8]. A variable scale fusion network model was employed with residual blocks and an attention mechanism to convert signals into a spectrogram [9]. Zhai [10] converted the signals into double-coupled matrices as two-dimensional feature inputs for a CNN model. However, in view of the time-frequency spectrogram, these methods may have difficulty in accurate modeling with information extraction from ECG signals with a low signal-to-noise ratio.

In order to address this issue, the thought of transfer learning was utilized to develop a deep CNN model, which converted signals into a two-dimensional recurrence plot [11]. This improves the ability of the recurrence plot to represent both temporal and spatial features of signals, improving the classification accuracy with an expression of multi-dimensional information. However, the dimensionality reduction parameter is a key determination for the recurrence plot. Thus, empirical judgment or multiple parameter attempts may be necessary to achieve relatively accurate results.

An improved ResGC-Net network for automatic arrhythmia recognition was employed by converting signals into a two-dimensional Gramian Angular Field (GAF) for identification and classification [12]. This method can better represent the interactions between different parts of signals, while possessing a superior stability and robustness. A semi-supervised CNN was proposed [13] for arrhythmia classification by using Markov Transition Field (MTF) to analyze signals at different time and frequency scales. The MTF exhibited a robustness with a reduction in the influence of noise through adjustment of the regularization parameter of the state transition matrix. However, calculations of GAF and MTF involve a high computational complexity. Therefore, it may result in an extended calculation cost or inaccurate results when dealing with large-scale time series data.

In automatic arrhythmia classification, a primary challenge is to exploit a simple and efficient rise-dimensional algorithm for the image format to support subsequent automatic classification tasks. To address this need, a novel technology is proposed that utilizes relative position matrices for feature extraction in processing signals. This approach enables the swift conversion of a two-dimensional image while retaining sufficient information to facilitate subsequent automatic classification tasks. The proposed method is incorporating a deep learning algorithm via the Gam-Resnet18 network, which is built upon ResNet, by introducing the GAM global attention mechanism to enhance the capability of feature selection. The target of the proposed technology is to achieve rapid and effective detection and diagnosis of arrhythmias.

The organization of this paper can be described as follows. The ECG signals for the abnormal heart rate types underwent segmentation firstly. Wavelet transform with a db6 wavelet and five-scale decomposition are applied to the segmented signals for noise reduction. In order to accurately represent the features of signals, the conversion of time-domain signals is conducted into relative position matrices in Section 3. Then, an in-depth explanation of the proposed network model “Gam-Resnet18” is provided. The design strategy using transfer learning technique is discussed, which is applied to facilitate automated classification. Section 5 concentrates on constructing a transfer convolutional neural network accounting for modeling of the features represented by two-dimensional images. It is demonstrated that this model achieves an overall accuracy of 99.30% in the context of relative position matrix-based classification. To validate the effectiveness of the proposed method, comparisons are also made with other algorithms using GAF, RP, and MTF.

## 2. Data Processing Method

### 2.1. Introduction of the Dataset

The present study involves the utilization of ECG signal recordings from the MIT-BIH Arrhythmia Database [14]. The data are acquired from a total of 47 participants. These samples include 25 male individuals aged 32 to 89 years, along with 22 female individuals aged 23 to 89 years. In order to facilitate the analysis, we opted to select all 38 records that featured MLII lead configurations. The selected heart rhythms from the samples include normal electrocardiograms (Normal), and four prevalent arrhythmia types (e.g., left bundle branch block (LBBB), right bundle branch block (RBBB), atrial premature contractions (APC), and premature ventricular contractions (PVC)). In the subsequent discussion, the designations N, L, R, A, and V are chosen to represent these five electrocardiograms.

### 2.2. ECG Signal Segmentation

In the procedure of diagnosing signals, it is necessary to perform the segmentation of heartbeats for the extraction of essential features. Due to the atypical waveform of arrhythmic signals, localization of ECG signals is required with precise identification of QRS peak positions. The MIT-BIH dataset has been completed using the manually labeled R-peak positions, thereby facilitating the subsequent process of heartbeat segmentation.

**Figure 1:**
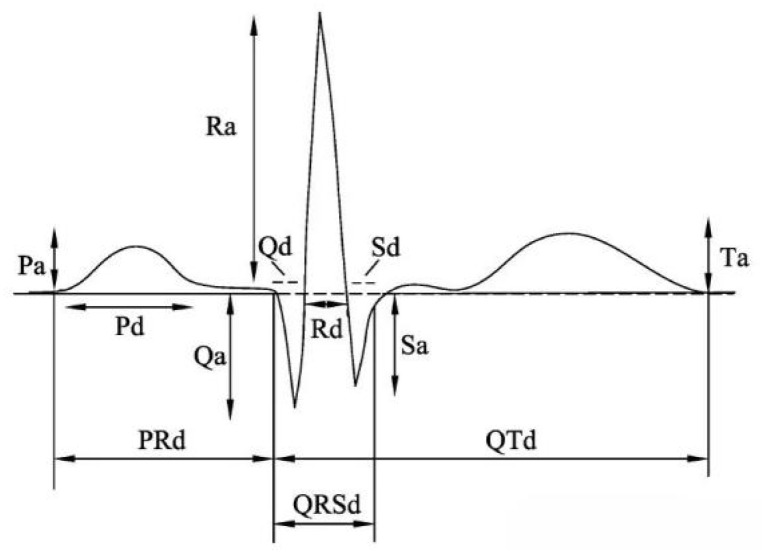
Ambulatory electrocardiogram.

Heartbeat segmentation is a key step in ECG signal processing. Commonly, heartbeat segmentation relies on fixed sampling points or time windows with QRS peak positions (Figure 1). Specifically, QRS peak locations serve as the starting point with 99 signal points forwards and 200 signal points backwards. Then, these points are compiled to be a complete heartbeat to guarantee the consistency and precision. With processing and filtering of the original ECG signals from MIT-BIH, all patients with the MLII lead are selected for research. Table 1 displays the number of samples for each type of ECG by applying the individual heartbeat segmentation. And it is noted that these are actual samples taken from the original database.

To address the issue of data imbalance in ECG signals, an approach is employed for individual normal heartbeats conversion of 5 s data segments. By converting the single normal heartbeat into segments of 5 s, the challenge of imbalanced data can be effectively tackled. Additionally, the exclusion of 6 or 7 s time intervals is taken into account to mitigate the impact of noise and interfering signals, thereby enhancing the accuracy and reliability in detecting specific cardiac events.

To perform the 5 s segmentation, it starts from the first sampling point at an interval of 5 s (1800 data points). In accordance with the established criteria, the segments will be labeled as designation of N if all the heartbeats within the segments correspond to the normal category. This approach is conducted to address the data imbalance caused by solely segmenting individual heartbeats, thereby enhancing the integrity and reliability of the dataset. Consequently, the particular outcome of the mixed heartbeat samples is referred to Table 2.

### 2.3. Denoising of Electrocardiogram Signals

The ECG is a weak biological electrical signal that is subject to various sources of interference. So, it is imperative to perform noise reduction processing in a targeted manner. In this paper, the db6 wavelet function is selected from the Daubechies wavelet family for suppressing noise. Within the support interval (−2, 2), it can be represented as a symmetric function centered at 0. The mathematical expression is defined as:

(1) $$\psi (t)=h_{0}*\varphi (t)+h_{1}\times \varphi (2t)+h_{2}\times \varphi (2t-1)+h_{3}\times \varphi (2t-2)+h_{4}\times \varphi (2t-3)+h_{5}\times \varphi (2t-4)$$

Here, *ψ*(*t*) represents the db6 wavelet function, *φ*(*t*) denotes the scaling function, and *h*_0_, *h*_1_, *h*_2_, *h*_3_, *h*_4_, and *h*_5_ are the coefficients of the db6 wavelet. The specific coefficients for the db6 wavelet are *h*_0_ = 0.332671999, *h*_1_ = 0.806891509, *h*_2_ = 0.459877502, *h*_3_ = −0.13501102, *h*_4_ = −0.085441273, and *h*_5_ = 0.035226293. These coefficients are derived to ensure that the db6 wavelet satisfies the conditions of compact support and orthogonality.

The db6 function is chosen here due to its similarity with the QRS waveform parameters found in ECG signals. Through the utilization of the wavelet transform, signals can be decomposed into wavelet coefficients of different scales and frequencies, providing a more precise description of characteristics. For ECG signals, the energy primarily converges within 4 or 5 scales. Accordingly, a decomposition level of 5 is chosen for multiscale analysis in this study. A balance could be achieved for a decomposition level of 5 between high frequency characteristics and noise suppression while fully retaining the low frequency information of the signal [15].

In wavelet denoising, hard and soft thresholding methods are employed typically. The compromise of soft–hard thresholding [16] aims to balance the advantage and limitation of both methods. Usually, soft thresholding is applied initially to eliminate noise. Subsequently, hard thresholding is used to remove the remaining noise while maximizing the retention of signal details. The threshold function of the approach is defined as follows:

(2) $$Y=\left\{\begin{array}{cc} sign(Y)\cdot (\left|Y\right|-\alpha \lambda ), & \left|Y\right|\geq \lambda \\ 0, & \left|Y\right|<\lambda \end{array}\right.$$

**Figure 2:**
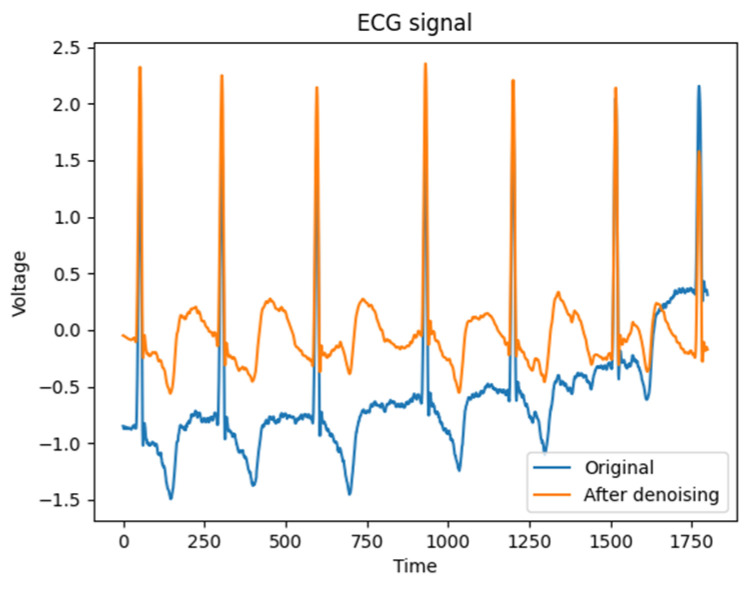
Comparison of ECG signal denoising.

When *λ* is assigned a value of 0 or 1, the threshold function manifests as either hard or soft thresholding, respectively. However, when 0 < *λ*< 1, both soft and hard thresholding methods are applied to the ECG signal. In this study, an intermediate weight of *α* = 0.5 is chosen as a compromise. The signal denoising is shown in Figure 2, which is obtained a good processing effect in practice.

## 3. Information Feature Extraction Strategy Based on Relative Position Matrix

The manual extraction of features from ECG is a complex and time-consuming task due to the large volume of data. Furthermore, a challenge is presented for CNN to accept inputs of different lengths of ECG recordings. The conversion of time-domain ECG data into two-dimensional images offers several advantages. It facilitates feature extraction, leading to improved performance. Neural networks are well suited for processing two-dimensional matrix-format data. The challenge of data with varying lengths can be overcome by normalizing through segmented aggregation approximation, enabling uniform conversion to images of consistent size. The Relative Position Matrix (RPM) [17] is a visualization method, which captures the relative positions between different moments in a time series. Furthermore, it enhances data interpretability by reflecting the relative position of each moment within the entire time series. The algorithm of RPM provides a more comprehensive characterization of the correlation and trend among different data points. The dependency relationship is reflected among various temporal moments within a time series.

**Figure 3:**
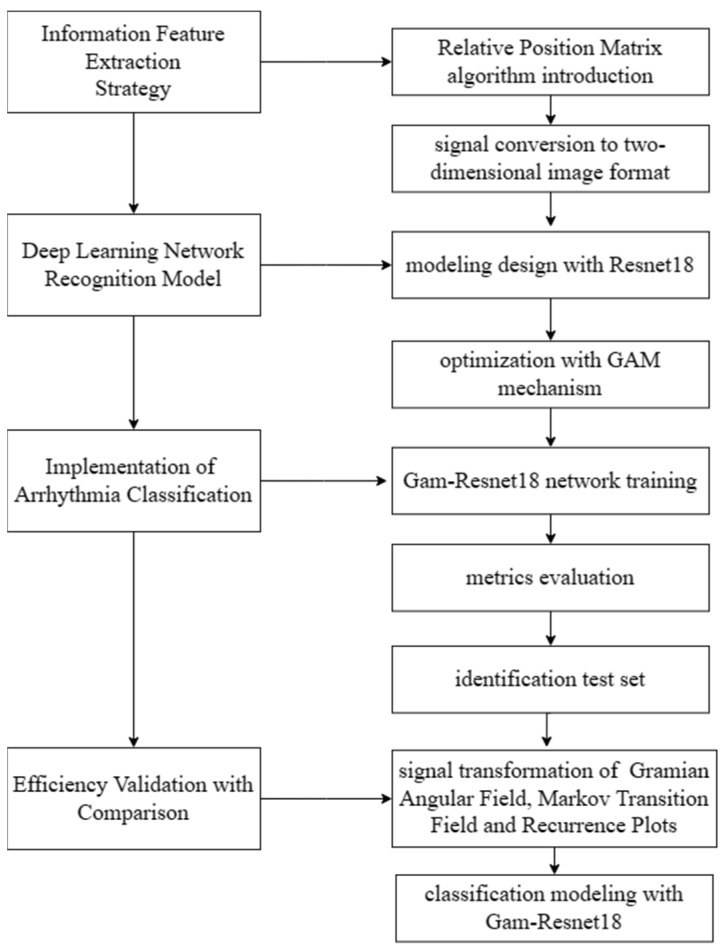
Design of the modeling process.

Therefore, RPM is proposed to convert ECG signals into two-dimensional images, facilitating a better feature extraction and accurate classification. The deep learning network modeling for recognition and classification is performed in subsequent sections. The detail of the modeling process is shown in Figure 3.

### 3.1. Relative Position Matrix Algorithm

The electrocardiogram signal is represented as *X* = *x*_1_, *x*_2_,…, *x_n_*, where x_i_ is the value at each sampling point at time step *i*, and the length of the signal is *n*. The RPM algorithm can be described as follows.

1. To obtain a standard normal distribution Z for the ECG signal. The z-score normalization can be performed as:
  (3) $${\tilde{x}}_{t}=\frac{x_{t}-\mu }{\sigma },\;t=1,2,\ldots ,n$$
  where *μ* is the mean of *X* and *σ* is its standard deviation.
2. Calculate the relative position between two time steps and transform the pre-processed ECG signal *X* into a two-dimensional matrix *M*. Each value at time step *i* serves as the reference point for each row of *M*. The transformation equation is formulated as follows:
  (4) $$M=\left[\begin{array}{cccc} {\tilde{x}}_{1}-{\tilde{x}}_{1} & {\tilde{x}}_{2}-{\tilde{x}}_{1} & \ldots & {\tilde{x}}_{m}-{\tilde{x}}_{1}\\ {\tilde{x}}_{1}-{\tilde{x}}_{2} & {\tilde{x}}_{2}-{\tilde{x}}_{2} & \ldots & {\tilde{x}}_{m}-{\tilde{x}}_{2}\\ \ldots & \ldots & \ldots & \ldots \\ {\tilde{x}}_{1}-{\tilde{x}}_{m} & {\tilde{x}}_{2}-{\tilde{x}}_{m} & \ldots & {\tilde{x}}_{m}-{\tilde{x}}_{m} \end{array}\right]$$

The resultant matrix *M* characterizes the relative position relationships between each pair of time steps in the ECG signal sequence. Each row and column of the matrix *M* is centered on a reference time step, further characterizing the information of the entire sequence. Each row of matrix *M* displays a time series with different reference points, while each column shows the mirror image of the former, providing a reverse perspective for viewing the time series.

3. The final gray-level matrix *F* is obtained by applying minimum–maximum normalization below:

(5) $$F=\frac{M-\min(M)}{\max(M)-\min(M)}\times 255$$

To effectively reduce the dimensionality of signals, the Piecewise Aggregate Approximation (PAA) method is adopted here. By calculating the average values of the piecewise constant to reduce dimensionality, the approximate trend of the original ECG signal can be maintained effectively. Ultimately, the smooth ECG signal sequence is transformed to the matrix representation of the two-dimensional image, denoted as matrix *F*.

### 3.2. Conversion of Relative Position Matrix ECG Image

**Figure 4:**
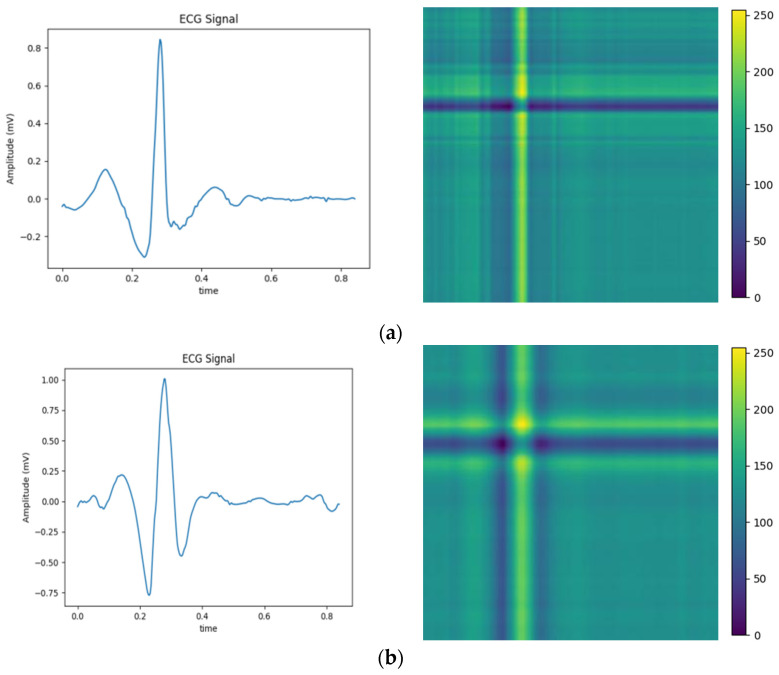

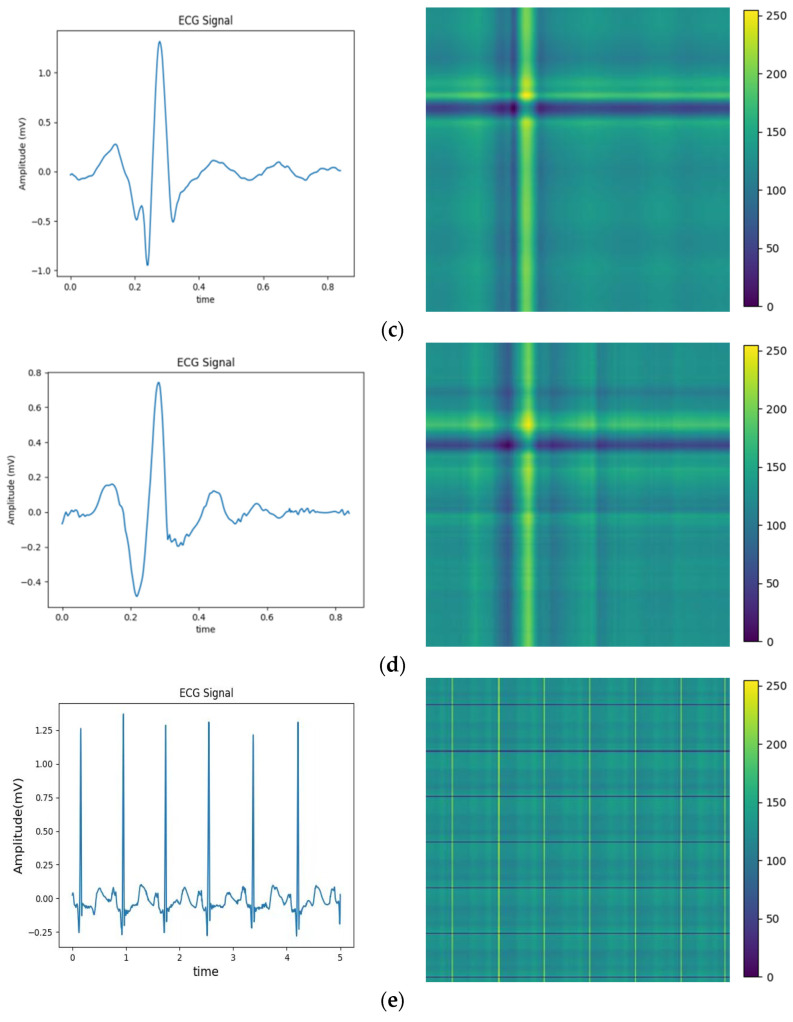
Conversion of five types of ECG signals in relative position matrix: (**a**) APC; (**b**) LBBB; (**c**) RBBB; (**d**) PVC; and (**e**) Normal ECG.

The above balanced segmented samples are converted into two-dimensional images using a relative position matrix in this part. In the course of the conversion, a pixel resolution can be set to control image accuracy and clarity, such as 224 × 224. The transformed images are generated based on the feature RPM obtained from preprocessing, as shown in Figure 4. The areas with high scales in the figure correspond to the locations with elevated amplitudes in the original ECG. To reduce the dimensionality of ECG samples for normal heartbeats, the PAA method is employed. Therefore, the dimensionality is reduced to 300 × 300, as the relative position map illustrated in Figure 4e. This method extracts an intuitive display of features and local structure in the ECG signal. It also provides important information for subsequent analysis and processing.

## 4. Design of Gam-Resnet18 Network Model for Relative Position Matrix Recognition

**Figure 5:**
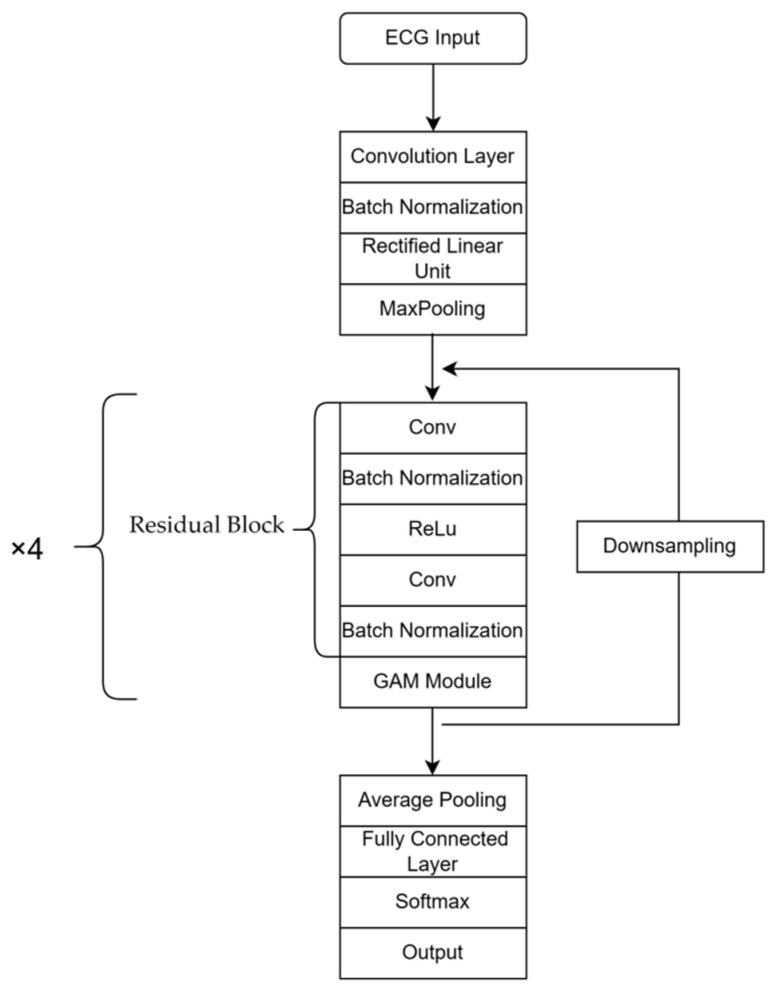
Network architecture diagram of Gam-Resnet18.

ResNet18 [18] is a deep learning model primarily utilized in the field of image classification. It comprises convolution and pooling layers, residual blocks and fully connected layers, which are used to extract image features and address issues of model degradation, respectively. In this study, the Gam-Resnet18 model is proposed as an improvement on ResNet18, specifically customized for the categorization of ECG signals. In the Gam-Resnet18 model, a GAM [19] module is added to enhance feature selection at the output of each residual block. This module performs global average pooling and uses a multi-layer perceptron to calculate the weights associated with each channel. Subsequently, the weighted feature map is then added to the original feature map to obtain an output with enhanced information features. Compared to ResNet18, the Gam-Resnet18 model shows improved feature selection ability due to the addition of GAM modules. The proposed model procedure is shown in Figure 5 for the structure of the network. In the procedure of modeling, the 2D convolution layer and max pooling layer are utilized to extract time series features from ECG signals, which are transformed into image format by the residual blocks and GAM modules.

## 5. Implementation of Arrhythmia Classification Based on Gam-Resnet18 Network

### 5.1. Gam-Resnet18 Network Training

The dataset is partitioned into separate training and test sets in an 8:2 ratio, with 20% of the training set reserved as a validation set. During the model training, an image dimension of 224 × 224 is utilized with a batch size of 32 and a learning rate at 0.0001. The network optimization is undertaken through the utilization of the Adam optimizer, which dynamically adjusts the updated step size for each parameter via adaptive learning rates. This approach is accelerated in the training process, avoiding the problem with local optimization. The performance of the model is evaluated by monitoring accuracy and loss value on the validation set.

**Figure 6:**
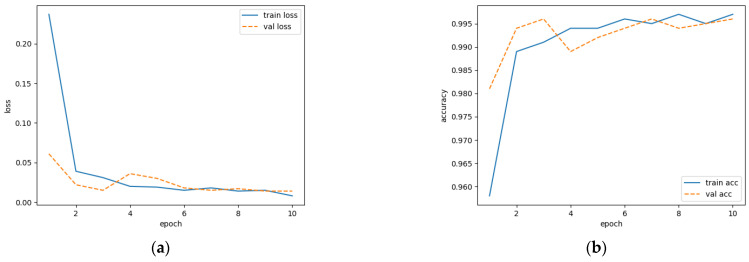
Modeling process of network training: (**a**) loss variation; (**b**) accuracy variation.

The loss value can be reflected by the discrepancy between the predicted result and the true label, while accuracy is measured by the proportion of correctly predicted samples to total samples. The training processes are shown in Figure 6. It is demonstrated that the loss value in the validation set is stabilized after the sixth epoch. The accuracy approaches the value from the training set after the ninth epoch. This finding indicates that the model has a strong generalization ability.

### 5.2. Metrics Evaluation

The performance evaluation framework is introduced for the image classification model, including five key metrics as overall accuracy (*ACC*), positive predictive value (*PPV*), specificity (*SP*), sensitivity (*SE*), and F1 score. Overall accuracy is represented by the proportion of correctly classified heartbeat signal samples to total samples, while *PPV* is the ratio of correctly classified positive samples to all positive samples. Specificity is used to measure the probability of true negative samples being correctly predicted as negative. Sensitivity is referred to as the probability of true positive samples being correctly predicted as positive. The F1 score is the harmonic mean of precision and recall. By selecting appropriate metrics, the accuracy as well as the robustness of classifiers can be improved to meet requirements. The computations can be carried out using the following formulas.

(6) $$ACC=\frac{TP+TN}{TP+TN+FP+FN},\;PPV=\frac{TP}{TP+FP},\;SE=\frac{TP}{TP+FN},\;SP=\frac{TN}{TN+FP},\;F1=\frac{2\cdot PPV\cdot SE}{PPV+SE}$$

Here, the samples of arrhythmia can be correctly identified by the classifier using *TP*, while *FP* represents the samples of other types that the classifier has incorrectly identified. *TN* indicates the number of samples of the current type incorrectly identified by the classifier as other types, while *FN* represents the number of samples not belonging to the current type incorrectly identified by the classifier as other types.

To assess the performance of the model, the Receiver Operating Characteristic (ROC) curve and Area Under the Curve (AUC) are utilized as evaluation metrics. The ROC curve provides a visual representation of the relationship between the true positive rate (*TPR*) and the false positive rate (*FPR*) at various thresholds. The *TPR* and *FPR* can be calculated by using the following formulas.

(7) $$TPR=\frac{TP}{TP+FN},\;FPR=\frac{FP}{FP+TN}$$

**Figure 7:**
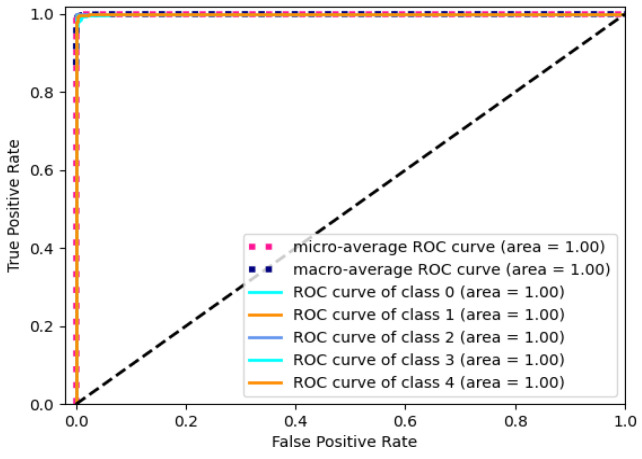
ROC curve of Gam-Resnet18 model with RPM.

The value of AUC ranging from 0 to 1 is adopted to quantify the discriminative ability of the model. A higher value indicates a better classification performance. The ROC curve of the model is presented in Figure 7 with the RPM. The black dashed line stands for the performance boundary between classifier and random selection. The curve approaching the upper-left corner states a better performance of the classifier for the corresponding category. The micro-average curve evaluates the overall performance by considering true positive and false positive rates across all categories. And, the macro-average curve represents the average value of the individual category curves.

The value of 1 for micro-average AUC signifies an excellent classification performance for the entire dataset. Similarly, the macro-average AUC of 1 indicates a good average classification performance across different categories. It indicates stable and accurate classification abilities across multiple categories. In summary, these results provide evidence of the outstanding performance of the Gam-Resnet18 model in classifying electrocardiogram signals.

### 5.3. Identification Results

**Figure 8:**
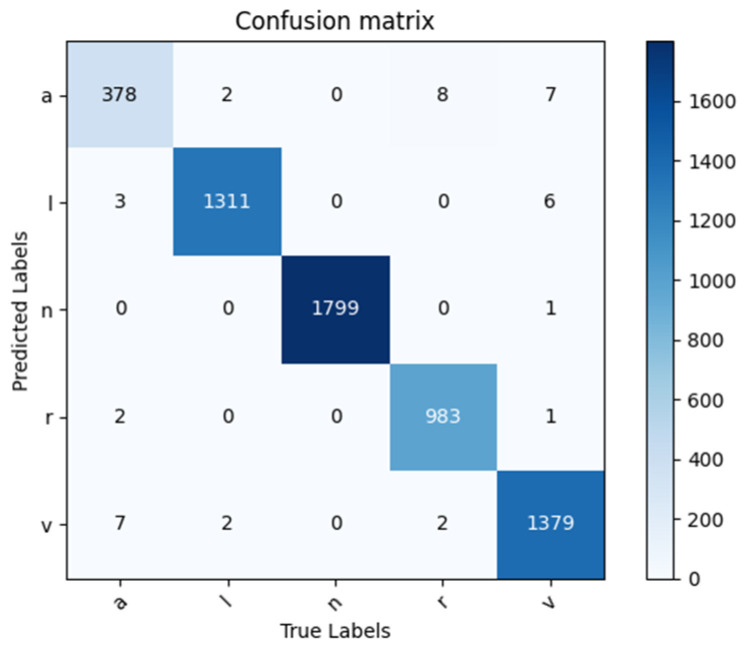
Confusion matrix of 5-class classification results.

By applying the trained model to the test data, the corresponding confusion matrix in Figure 8 is obtained, which can assess the classifier’s performance. Based on the confusion matrix, the classification evaluation indicators are calculated subsequently, which are shown in Table 3. The trained model demonstrates a perfect performance in classifying the five types of ECG signals with a classification accuracy rate of 99.30%. It indicates that this model can accurately classify ECG signals while distinguishing between different types of signals.

**Figure 9:**
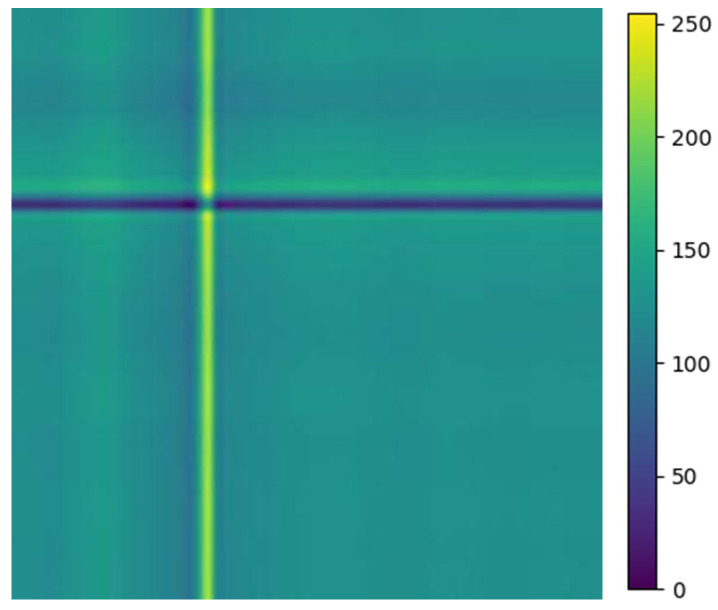
ECG normal image of relative position matrix for single heartbeat.

For the comparison of influence under heartbeat segmentation, the performance is achieved in ECG signal classification with the single heartbeat. As indicated in Figure 9, the normal image of RPM for the single beat is much neater compared to the arrhythmia types. The metrics in Table 3 reveal that a better performance is achieved when considering the mixed heartbeat for modeling. These findings highlight the enhanced validity of hybrid heartbeat segmentation in the RPM + Gam-Resnet18 model, making a reliable option for accurately classifying ECG signals.

## 6. Comparison of Images for Classification Modeling with Gam-Resnet18 Network

### 6.1. Transformation of ECG Signals

**Figure 10:**
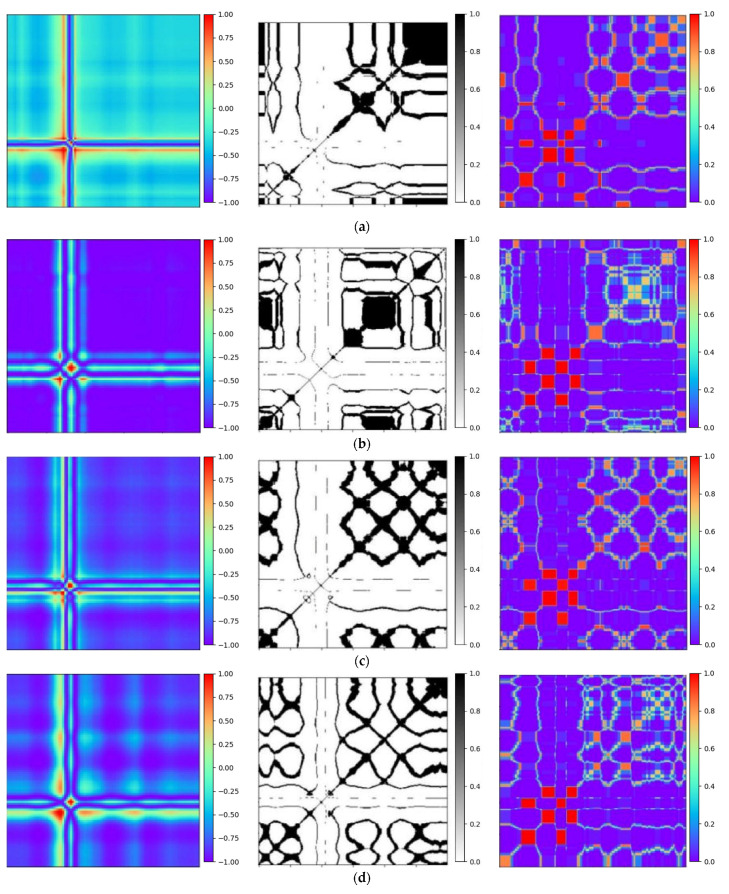

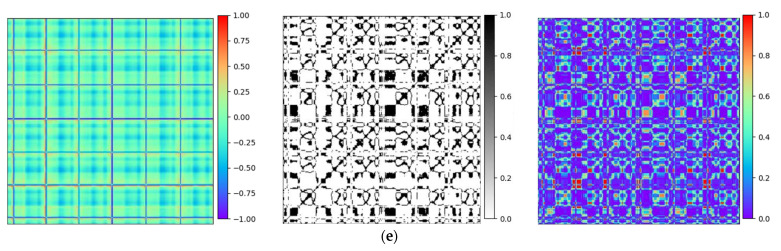
Transformation diagrams to Gramian Angular Fields, Recurrence Plots, and Markov Transition Fields: (**a**) APC; (**b**) LBBB; (**c**) RBBB; (**d**) PVC; and (**e**) Normal ECG.

To validate the efficiency of the proposed technique, Gramian Angular Field [20] is transformed for the identical ECG signal, as well as Recurrence Plots [21] and Markov Transition Field [20] simultaneously. Subsequently, verifications are performed by using the Gam-Resnet18 network model for the recognition of all image features. To ensure the consistency of the measurements in the experiment, a consistent pixel resolution of 224 × 224 is maintained with all samples and employed identical ECG signals. After data preprocessing, the corresponding GAF, RP, and MTF are generated, as illustrated in Figure 10. Three kinds of image formats are represented from left to right in each group of arrhythmias, which are derived from the GAF, RP and MTF, respectively. A direct correlation is expressed between color intensity and value magnitude in the GAF images. This correlation becomes even more pronounced as the color intensity increases. In the RP features, the black scale signifies the similarity among corresponding points, whereas the white scale indicates the dissimilarity. For the MTF images, the color scale corresponds to the heightened frequency of neighboring signals.

### 6.2. Modeling Results

During the model training process, identical hyperparameters are implemented including a batch size of 32 and a learning rate of 0.0001. Network optimization is utilized by the Adam optimizer. Model performance is also evaluated by monitoring the precision rate and loss value of the validation set. By employing the trained model on testing, the performance of classifier can be evaluated by acquiring corresponding confusion matrices. Based on these matrices, the overall accuracy rates are calculated as 99.15%, 99.28%, and 98.57% for the RP, GAF and MTF, respectively. Moreover, with a comparison to the relative position matrix algorithm, it is shown in Table 3 and Table 4 that the latter achieves a higher overall accuracy rate of 99.30%. An excellent performance is presented for the RPM algorithm in accurately classifying ECG signals. The results also confirm the reliability and effectiveness of this proposed method, offering a significant reference for ECG signal classification.

### 6.3. Comparison with the Original ECG Signal

In this section, a detailed comparison is conducted between RPM and the original ECG signal by employing the Gam-Resnet18 model. To ensure a fair comparison, the dataset is partitioned and trained using the same methodology. Furthermore, it is illustrated that an overall accuracy of 99.27% is achieved for ECG signal classification on the testing set. Comparatively, the RPM algorithm exhibits a higher overall accuracy of 99.30%, as shown in Table 3 and Table 5. This again indicates the effectiveness of the RPM + Gam-Resnet18 model on classifying cardiac arrhythmias.

### 6.4. Comparison with Reported Results

The RPM algorithm is compared with existing techniques as illustrated in Table 6 in this section. For instance, a transfer learning network based on AlexNet [22] was utilized to transform ECG signals into grayscale images, achieving a classification accuracy of 94.95%. Similarly, the particle swarm optimization [23] was employed with a support vector machine model. It achieves an accuracy rate of 98.57% for five-class classification. One-dimensional CNN model with LSTM was designed [24] to classify similar types of heartbeats, resulting in an overall accuracy of 98.10%. Additionally, the feature extraction method in combination with empirical mode decomposition (EEMD) was proposed [25] for classification with a sequential minimal optimization support vector machine (SMO-SVM). Compared to these results, the methodology of the proposed relative position matrix with Gam-Resnet18 network produces a higher classification accuracy, confirming its superiority for ECG signal classification.

### 6.5. Discussions

In future research, this method can be expanded and improved in several ways. Firstly, it can enhance the feature extraction capability of ECG signals by the improvement of the relative position matrix. Additionally, the integration of sophisticated data augmentation methods, such as the GAN (Generative Adversarial Network), could be employed to expand the dataset and enhance the robustness of the model. Thirdly, the classification accuracy can be further improved by combining adaptive learning and model fusion technologies. Finally, for noisy and anomalous data, more effective denoising and filtering methods may be employed to increase the model robustness and boost the classification performance.

## 7. Conclusions

A novel approach is presented in this paper to classify ECG signals using deep learning networks. To address the recognition strategy, a relative position matrix is utilized to describe the relative spatial relationships between different waveforms with Gam-Resnet18 network image recognition technology for classification. The goal of this method is to enhance the ability of feature extraction and classification accuracy of ECG signals by employing image transformation, attention mechanisms, and residual blocks. The main conclusions are summarized as follows.

In terms of data processing, ECG signals are segmented into the mixed heartbeat samples comprising single heartbeats and 5 s fragments. The segmented signals are denoised using the db6 wavelet basis and 5-level decomposition with wavelet transform. For image conversion, a feature extraction strategy is adopted based on a relative position matrix. This method extracts an intuitive display of features and local structure in the ECG signal, providing a support for automatic classification tasks.

To achieve the two-dimensional image recognition, a Gam-Resnet18 network model is proposed for the modeling and classification of ECG signals. The GAM global attention mechanism is introduced at each residual block to improve the recognition capability of the model. Additionally, transfer learning technology is employed to accelerate the model training process.

Utilizing the RPM approach with the Gam-Resnet18 modeling, the proposed method is validated with a total accuracy of 99.30%. The results are also compared with methods of GAF, RP, MTF, and other reported results to validate the effectiveness. It is demonstrated that the relative position matrix can better reflect the differences between various types of arrhythmias, thereby improving the accuracy and stability of classification.

## Figures and Tables

**Table 1:** Classification results of five types of heart rate based on single beat.

Heart Rate Types	A	V	L	R	N
Number	1950	6974	6578	4967	71,723

**Table 2:** Segregation results of the mixed heartbeat samples.

Heart Rate Types	A	V	L	R	N
Number	1950	6974	6578	4967	8996

**Table 3:** Performance metrics for 5-class classification with RPM.

Segment	Type	ACC	PPV	SP	SE	F1 Score	Average PPV	Average SE	Average SP
Mixed heartbeat	A	99.30%	95.7%	99.7%	96.9%	96.3%	98.76%	99.84%	98.90%
	L		99.3%	99.8%	99.7%	99.5%			
	N		99.9%	100.0%	100.0%	99.9%			
	R		99.7%	99.9%	99.0%	99.3%			
	V		99.2%	99.8%	98.9%	99.0%			
Single beat	A	99.07%	97.8%	99.9%	82.3%	89.4%	98.74%	99.30%	95.22%
	L		98.6%	99.8%	99.2%	98.9%			
	N		99.2%	97.1%	99.8%	99.5%			
	R		99.7%	99.9%	99.0%	99.3%			
	V		98.4%	99.8%	95.8%	97.0%			

**Table 4:** Evaluation metric results for cardiac arrhythmia classification.

Method	Type	ACC	PPV	SP	SE	F1 Score	Average PPV	Average SE	Average SP
RP	A	99.15%	94.9%	99.6%	96.2%	95.5%	98.50%	98.72%	99.64%
	L		99.0%	99.7%	99.8%	99.4%			
	N		100%	100%	100%	100%			
	R		99.3%	99.9%	99.5%	99.4%			
	V		99.3%	99.8%	98.1%	98.7%			
GAF	A	99.28%	96.2%	99.7%	97.4%	96.8%	98.80%	98.89%	99.82%
	L		99.0%	99.7%	99.5%	99.2%			
	N		100.0%	100.0%	100.0%	100.0%			
	R		99.5%	99.9%	99.4%	99.4%			
	V		99.3%	99.8%	98.6%	98.9%			
MTF	A	98.57%	95.0%	99.7%	92.3%	93.6%	97.92%	97.62%	99.68%
	L		98.6%	99.6%	99.2%	98.9%			
	N		99.9%	100.0%	99.9%	99.9%			
	R		98.5%	99.7%	99.1%	98.8%			
	V		97.9%	99.4%	97.6%	97.7%			

**Table 5:** Performance metrics for 5-class classification with original ECG signal.

Type	ACC	PPV	SP	SE	F1 Score	Average PPV	Average SP	Average SE
A	99.27%	96.2%	99.7%	97.7%	96.9%	98.76%	99.80%	99.02%
L		99.5%	99.8%	98.9%	99.2%			
N		99.9%	100%	100%	100%			
R		99.3%	99.8%	99.6%	99.4%			
V		98.9%	99.7%	98.9%	98.9%			

**Table 6:** Comparison of results with other literature.

Method	Type	ACC	SE	SP	PPV
AlexNet-like + Grayscale Image [22]	5	94.95%	-	-	-
LibSVM [23]	5	98.57%	-	-	-
CNN + LSTM [24]	5	98.10%	97.50%	98.70%	-
EEMD + SMO-SVM [25]	5	99.20%	98.01%	99.49%	-
RPM + Gam-Resnet18	5	99.30%	98.90%	99.84%	98.76%

## Data Availability

Not applicable.
